# Research Ambassador Program: An innovative educational approach to addressing underrepresentation of minority populations in clinical research

**DOI:** 10.1017/cts.2022.496

**Published:** 2022-11-09

**Authors:** Nicole Wolfe, Mayra Rubio-Diaz, Alma Garcia, Sara Calderon, Michele D. Kipke

**Affiliations:** 1 Southern California Clinical and Translational Science Institute, University of Southern California, Los Angeles, CA, USA; 2 Children’s Hospital Los Angeles, Los Angeles, CA, USA

**Keywords:** Clinical research, community engagement, education, community health workers, health disparities

## Abstract

Clinical trial participation among historically underrepresented populations remains low in large part due to mistrust of academic institutions and research investigators. Mistrust may be ever greater today given misinformation related to COVID-19. The Research Ambassador Program is an interactive educational workshop delivered by *Promotoras de Salud*/Community Health Workers and designed to both address common myths, fears, and concerns about research and encourage research participation among underrepresented populations. An evaluation conducted with 819 Latino and Black participants demonstrated a change in behavior and intention to participate in a clinical trial, with half of participants enrolling in a clinical trial research registry.

## Introduction

Racial and ethnic minorities comprise one-third of the population yet have not been proportionally represented in clinical research [1]. Clinical trial participation is essential to preventing, diagnosing, and understanding disease progression, to develop effective treatments to improve the health of individuals. Underrepresentation of minority populations has been identified as a barrier to advancing medical discoveries and promoting health equity, as racial and ethnic populations do not metabolize or respond to medications in the same ways [2,3]. Minority populations are up to two times more likely than their white counterparts to have most of the prevalent major chronic diseases [4], yet they comprise about only 23% of clinical trial participants [5], which may contribute to preventable disparities in treatment outcomes and survival rates for these populations.

To address our national goal of “achieving health equity, eliminating disparities, and improving the health of all groups” [6], innovative approaches are needed to fully engage diverse communities in the design, conduct, and dissemination of clinical and translational research. The challenge lies in developing community-partnered approaches that are both culturally relevant and which can also be evaluated and broadly implemented for widespread adoption. One of the leading barriers to research participation among racial and ethnic minorities is mistrust of academic institutions and research investigators [7,8]. The COVID-19 pandemic has exacerbated existing public mistrust in science, particularly among minority populations [7,9,10].

One approach to addressing this mistrust is the use of *Promotoras de Salud/* Community Health Workers who can be effective partners in community-based, public health research, and promotion due to their close relationships in their communities [11,12]. *Promotoras* are trained Latina community health workers who work directly in communities and are trusted by community members [12]. *Promotoras* have been a part of a variety of health promotion programs in the United States and have been successful in changing community knowledge and influencing behaviors around a variety of health issues [13–15].

To combat mistrust and encourage clinical trial participation among historically underrepresented populations, our Southern California Clinical and Translational Science Institute (SC CTSI) partnered with other Clinical and Translational Science Award (CTSA) hubs, local community organizations, and *promotoras* to develop an interactive educational workshop, called the Research Ambassador Program (RAP). This program was designed to empower members of the lay community from underrepresented populations to play an active role in clinical research, including clinical trials both as a community advisor and a research participant. The workshop covered a range of topics including an explanation about clinical research itself and the types of studies that fall under this umbrella; the history of clinical research; the experience of minority populations in clinical research; human subject research protection; and addressing myths and misinformation, to ultimately encourage participation in clinical research.

## Methods

### Description of the RAP

The RAP is a 90-minute educational workshop initially developed for Spanish-speaking Latino populations, which was subsequently adapted by our Community Health Workers for Black populations in recognition of the underrepresentation of both populations in clinical research and the need for education across those communities.

There were four primary areas of focus for this curriculum. First, address the myths, barriers, and fears about medical research. Second, provide a history of minority populations’ experience in the healthcare system and in medical research. This involved creating two separate presentations for the Latino populations and for the Black populations to address their unique historical experiences in medical research. Third, increase scientific literacy about how clinical research is conducted. Fourth, inform about the protection of human subjects in research and potential participants’ individual rights as a research participant.

The primary purpose of the RAP was to conduct culturally competent outreach in Latino and Black communities to educate and promote participation in clinical research, with the goal of reducing health disparities among minority populations in Southern California through the integration and increased participation of underrepresented groups in clinical research. The RAP was designed to be delivered by *promotoras/*Community Health Workers. It can be delivered in both English or Spanish, and either virtually or in-person. The *promotoras/*Community Health Workers who delivered these workshops were employees of the Community Engagement core group at the SC CTSI who have extensive experience in community education. They were trained to give these workshops through both their participation in the development of the workshop and the accompanying slide presentation, as well as by presenting to our team prior to delivering the workshop to community members. While they all followed the same presentation, it is acknowledged that we did not account for any potential differences in outcomes due to individual presentation styles.

### Implementation of the RAP

Participant outreach was conducted by *promotoras* in the geographically defined areas of the Eastside and South Los Angeles, communities in which our team worked and engaged. Working in pairs, they walked through those neighborhoods, visiting local parks, community centers, schools and parent centers, and churches speaking directly with staff and community members at those locations to recruit a range of community members. *Promotoras* attended and presented at school and church meetings to discuss and recruit for these workshops, which provided them the opportunity to describe the RAP and to answer any questions directly. Access to school principals, who approved their attending school meetings, came through the parent coordinators at the parent centers, which have significant engagement in the Latino community. As a result, many of the participants were church members and/or parents of children attending the local schools. While there was not a specific intent to recruit church members or parents, those two institutions proved to be the most effective arenas for recruitment due to the large numbers of community members who congregate at both.

*Promotoras* obtained individual’s contact information at the conclusion of their presentations and followed up directly to schedule each person for a workshop at a time of their convenience. Verbal consent was given twice by each participant, once at the initial meeting when the individual signed up for the workshop and again on the date of the workshops. In each instance, time was allotted for questions. All research protocols were approved by the University of Southern California’s Institutional Review Board (IRB: HS-17-00030).

## Results

Sixty-five 90-minute interactive, in-person workshops were conducted between 2017 and 2020, with a total of 819 participants. The interactive nature of the workshops allowed for dialog between the participants and the *promotoras* and for questions to be answered in real time. There was an average of 10 people per workshop, and the workshops were conducted in Spanish for the Latino participants and English for the Black participants. The locations included local schools, churches, recreation centers/gyms, and a public park in the communities from which the participants were recruited. As the workshop was initially created for the Latino population, there were a significantly larger number of Latino participants than Black participants with 703 and 106, respectively. The complete demographics are presented in Table 1 and show that 90% of the participants were female. More than half of the participants had an income level under $20,000 a year (58%) and had not completed high school (52%). Most participants had health insurance (76%) and a regular place to receive medical care (83%).

**Table 1.** Demographic information n = 819 (%)

Gender	
Female	740 (90)
Male	74 (9)
Transgender	1 (<1)
No response	4 (<1)
**Age**	
18–25	40 (5)
26–35	128 (16)
36–45	260 (32)
46–55	182 (22)
56+	206 (25)
No response	3 (<1)
**Race/Ethnicity**	
Latino	698 (85)
Black	106 (13)
White	8 (<1)
Asian	2 (<1)
Multiracial	2 (<1)
No response	3 (<1)
**Income per year**	
<$20,000	476 (58)
$20,000–$29,999	151 (18)
$30,000–$39,999	87 (11)
$40,000–$49,999	39 (5)
$50,000–$74,999	16 (2)
>$75,000	22 (3)
No response	28 (3)
**Education**	
<High school	429 (52)
High school/GED	228 (28)
Some college	97 (12)
Bachelor’s degree	25 (3)
Graduate degree	18 (2)
No response	22 (3)
**Marital status**	
Single	257 (31)
Married/partnered	477 (58)
Divorced	37 (5)
Widowed	36 (4)
No response	12 (2)
**Language**	
English	188 (23)
Spanish	602 (73)
English/Spanish	14 (2)
No response	15 (2)
**Has Health Insurance**	
Yes	623 (76)
No	180 (22)
No response	16 (2)
**Has a Place to Receive Medical Care**	
Yes	682 (83)
No	121 (15)
No Response	16 (2)

Table 2 presents the number of workshops held between 2017 and 2020. The workshops began in 2017 with a total of 17 workshops held for Latino participants. By 2019, the workshop had been modified and expanded to include Black participants. Recruitment increased among both populations and in that year, 42 workshops were held. In 2020, six workshops were held before they were put on hold due to the COVID pandemic.

**Table 2.** Number of Workshops

Year	Total Number of workshops	Number for Latino Participants	Number for Black Participants
2017	17	17	0
2019	42	34	8
2020	6	4	2

Our research shows that people were more willing to enroll in a clinical trial registry following participation in the RAP workshops. These workshops intended to increase knowledge about clinical research and influence behavior among both Latino and Black populations. As Table 3 indicates, participation and completion of the workshop significantly impacted people’s intention to participate in a clinical trial, resulting in roughly half of participants (49%) enrolling in a clinical trial research registry, which was our primary outcome. We did not include any formal measurements of trust or clinical research knowledge. This was consistent across both Latino (49%) and Black (46%) populations and between females (47%) and males (59%).

**Table 3.** Enrolled in Trial Registry

	n (%)
**Latino (699 Total)**	
Female (646)	Female = 311 (48)
Male (53)	Male = 34 (64)
	Total = 345 (49)
**Black (106 Total)**	
Female (85)	Female = 39 (46)
Male (21)	Male = 10 (48)
	Total = 49 (46)
**Total (814)** *	
Female (740)	Female 350 (47)
Male (74)	Male 44 (59)
	Total = 392 (48)

* This number differs from the 819 total participants because this is counting only those who identified as either male or female and either Latino or Black

## Discussion

Clinical trial research conducted with diverse populations is essential to both improving the health of our nation and addressing health disparities and inequities. Building these partnerships and creating strategies to increase trust in science and increase the participation of underrepresented populations in clinical research is the call and the challenge to the Translations Sciences. This would help to ensure that discoveries, interventions, and prevention strategies can be generalized across societal populations. The evidence is clear that representation is critical and in this current climate where medical research and advances in medicine have become polarized and politicized, the urgency is even greater.

Meeting people where they are is the key to reaching them. The use of *promotoras/*Community Health Workers can be an effective approach to addressing the trust barrier that exists among Latino and Black populations by providing more transparency about the research process. The RAP leveraged the relationships between *promotoras*/Community Health Workers and the communities in which they are engaged and aimed to educate, engage, and empower diverse, underrepresented populations to be active participants in research with the ultimate goal to contribute to medical knowledge and advancement. This work adds a new element to this discussion through our focus on diversity, equity, and inclusion.

The current climate of mistrust in academic institutions and medical research must be recognized and addressed to avoid negative impact to researcher’s ability to enroll minority participants in clinical research, which directly impacts the ability to develop culturally tailored treatments/interventions [16]. Now more than ever, it is important to re-establish that trust to regain ground in clinical trials to positively impact the future of medical research. The challenge then is to build strong academic–community partnerships to provide information, engage, and empower individuals. This RAP is one approach to connecting with these communities to address the myths and misinformation around clinical research and to reinforce the importance of participation. While this was developed prior to the COVID pandemic, the need to engage in these types of approaches is even greater now.

The effectiveness of the RAP is evident in the change in behavior seen in the participant’s enrollment in the trial registry and willingness to participate in research. It is acknowledged that participants enrolled in a registry following the workshop, which included compensation of $40, so it is unknown how many would have chosen to enroll outside of that context, or if the compensation influenced that decision. Additionally, enrolling in a registry does not guarantee enrolling in a trial, and we do not have data to know how many people ultimately enrolled in a clinical trial.

Our findings suggest that the use of *promotoras/*Community Health Workers can increase community knowledge about, and intent to participate in, clinical research. We restarted the RAP in the fall of 2022 under a new IRB and have included a pre–post survey to assess a change in knowledge as well as behavior, and a satisfaction survey to be responsive to participant feedback and interest. The intent is that this program will increase in breadth and scope to continue to educate underrepresented populations to increase participation in medical research and ultimately improve health outcomes across those populations.

Underrepresentation is broader than just racial and ethnic groups and includes those across the age spectrum as well as sexual and gender minorities [17,18]. A robust clinical research infrastructure is needed to ensure this level of inclusion. The need for and use of medical interventions cuts across all demographic lines and as such, research should include as wide a range of people as possible to ensure maximum effectiveness of those interventions. This is what we suggest, and this is the direction that we, as a society, as researchers, and medical professionals, need to be moving.
